# Production of Recombinant Disulfide-Rich Venom Peptides for Structural and Functional Analysis via Expression in the Periplasm of *E. coli*

**DOI:** 10.1371/journal.pone.0063865

**Published:** 2013-05-07

**Authors:** Julie K. Klint, Sebastian Senff, Natalie J. Saez, Radha Seshadri, Ho Yee Lau, Niraj S. Bende, Eivind A. B. Undheim, Lachlan D. Rash, Mehdi Mobli, Glenn F. King

**Affiliations:** Institute for Molecular Bioscience, The University of Queensland, St. Lucia, Australia; Centre National de la Recherche Scientifique, Aix-Marseille Université, France

## Abstract

Disulfide-rich peptides are the dominant component of most animal venoms. These peptides have received much attention as leads for the development of novel therapeutic agents and bioinsecticides because they target a wide range of neuronal receptors and ion channels with a high degree of potency and selectivity. In addition, their rigid disulfide framework makes them particularly well suited for addressing the crucial issue of *in vivo* stability. Structural and functional characterization of these peptides necessitates the development of a robust, reliable expression system that maintains their native disulfide framework. The bacterium *Escherichia coli* has long been used for economical production of recombinant proteins. However, the expression of *functional* disulfide-rich proteins in the reducing environment of the *E. coli* cytoplasm presents a significant challenge. Thus, we present here an optimised protocol for the expression of disulfide-rich venom peptides in the periplasm of *E. coli*, which is where the endogenous machinery for production of disulfide-bonds is located. The parameters that have been investigated include choice of media, induction conditions, lysis methods, methods of fusion protein and peptide purification, and sample preparation for NMR studies. After each section a recommendation is made for conditions to use. We demonstrate the use of this method for the production of venom peptides ranging in size from 2 to 8 kDa and containing 2–6 disulfide bonds.

## Introduction

Animal venoms are gaining increased attention as a source of novel bioactive peptides [1], [2]. The venoms of arthropod predators such as spiders, scorpions, and centipedes are essentially combinatorial peptide libraries that have been optimised for high potency and selectivity against their molecular targets over hundreds of millions of years of evolution [3]. These biochemical attributes have allowed some receptors to be pharmacologically characterized via their interaction with various animal toxins. Moreover, the inherent stability, specificity and potency of disulfide-rich venom peptides have made them an attractive source of lead compounds for the development of new therapeutic agents and bioinsecticides [2], [4], [5].

One of the major challenges when working with venom peptides is to obtain sufficient material for structural and functional characterisation. This is especially problematic when working with disulfide-rich venom peptides that may form non-native disulfide-bond isoforms [6]. For example, a peptide with three or four disulfide bonds could theoretically form 15 or 105 different disulfide-bond isomers, respectively.

Historically, most venom components were obtained via purification from native material [7]. This approach is only viable for abundant components and even then the small amount of final product often limits the amount of structure-function characterization that is possible. More recently, solid-phase peptide synthesis (SPPS) has become the dominant means by which venom peptides are produced [8]–[10]. This approach has the advantage of allowing the introduction of non-native amino acid residues and posttranslational modifications. However, in most cases, this approach necessitates extensive screening of *in vitro* folding conditions and it therefore remains an expensive means of producing venom peptides [8]. A less costly approach is recombinant production of venom peptides in a suitable host. The Gram-negative bacterium *Escherichia coli* has long been an attractive host for heterologous protein expression [11]. Heterologous proteins are generally expressed in the cytoplasm of this bacterium as it offers the advantage of high protein yields and simple plasmid constructs. However, a major challenge with intracellular expression of disulfide-rich peptides in *E. coli* are the low yields of correctly folded (native) protein due to the reducing environment in the intracellular space [11]. If allowed to accumulate within the cytoplasm, recombinant proteins are often sequestered into aggregates known as inclusion bodies. Functional protein can be recovered using denaturant-induced solubilization, followed by optimization of refolding conditions [12]. This is often a laborious process, especially for disulfide-rich peptides, and finding a folding condition that will give high yield of the native fold is not guaranteed. Several approach have been introduced to make the cytoplasm of *E. coli* more suitable for expression of disulfide-rich proteins. These include making the cytoplasm less reducing by introducing mutations into the genes encoding glutathione reductase (*gor*) and thioredoxin reductase (*trxB*) (e.g. Origami™ strains) and by introducing a cytoplasmic disulfide isomerase protein (*DsbC*) to enhance disulfide bond formation (e.g. Shuffle™ strain).[13] An alternative approach to overcoming these problems is to bypass the cytoplasm altogether and have the nascent protein secreted into the periplasm of the bacterium, where the endogenous protein machinery for disulfide bond formation is located [14]–[16]. In essence, this allows one to hijack the existing *E. coli* refolding machinery in order to produce heterologous peptides with their native disulfide-bond arrangement.

The ability to produce recombinant disulfide-rich peptides in *E. coli* is not only cost effective, but it has the added benefit of enabling isotopic labelling of peptides for multidimensional, heteronuclear NMR studies [17]. NMR is the dominant approach for solving the structure of proteins smaller than 10 kDa, with ∼80% of all structures of peptides <5 kDa having been solved using this approach [1], [17]. Although homonuclear NMR approaches can be used to solve the structure of unlabelled peptides, the precision and stereochemical quality of the structure is generally better if the peptides are uniformly labelled with ^15^N and ^13^C and subjected to 3D/4D heteronuclear NMR experiments [17], [18]. Isotopic labelling also facilitates study of the dynamic properties of the peptide [19], [20].

Here we present a nine-step protocol for obtaining correctly folded disulfide-rich peptides for functional and structural characterization. This protocol is based on our experience in production of recombinant disulfide-rich venom peptides. Table 1 outlines the range of peptides that have been expressed using this system, which includes peptides ranging in size from 2 to 8 kDa and containing 2–6 disulfide bonds. The table includes both successful and failed attempts and reveals an overall success rate of 75%. Table 1 also includes several biophysical properties that may affect protein expression and folding but within this group of proteins no general trends can be discerned.

**Table 1:** Summary of the diverse range of disulfide-rich venom peptides produced in our lab using periplasmic expression.

Toxin name	Organism	No. of residues	No. of SS bonds	Yield of correctly folded peptide^1^	No. of contiguous hydrophobic residues [total]	No. of charged residues [net charge]	pI^2^	Isoforms^3^	Ref.
U_2_-CUTX-As1a	spider	37	4	++	2 [7]	5 [+1]	7.69	2	[91]
DkTx	spider	76	6	+	4 [23]	22 [+2]	6.84	3	[92]
OAIP 1	spider	33	3	++	2 [6]	11 [+5]	8.35	1	[93]
OAIP 2	spider	33	3	++	2 [10]	8 [+4]	8.64	1	[93]
OAIP 3	spider	34	3	++	3 [9]	5 [+1]	7.77	1	[93]
U_1_-AGTX-Ta1a	spider	51	3	+++	3 [15]	9 [−1]	5.14	1	[94]
U_1_-CUTX-As1c	spider	76	4	*—*	4 [19]	14 [+4]	8.15	*—*	[95]
U_1_-PLTX-Pt1a	spider	46	5	*—*	2 [10]	9 [−1]	5.02	*—*	[96]
U_1_-TRTX-Pc1a	spider	33	3	++	3 [12]	5 [+4]	8.35	1	[97]
U_2_-SGTX-Sf1a	spider	46	4	+++	3 [13]	9 [+1]	6.01	1	[90]
U_2_-TRTX-Pc1a	spider	28	3	++	1 [5]	5 [+5]	9.22	1	[97]
β-TRTX-Cm1b	spider	33	3	+	2 [8]	12 [+4]	8.86	1	[98]
β-TRTX-Ps1a	spider	34	3	+++	3 [9]	10 [+4]	8.89	1	[98]
β/ω-TRTX-Tp1a	spider	35	3	*—*	3 [10]	8 [+4]	8.37	*—*	[99]
κ-TRTX-Gr3a	spider	34	3	+++	3 [11]	9 [+3]	8.66	1	[100]
κ-TRTX-Tb1a	spider	35	3	*—*	2 [9]	12 [+4]	8.64	*—*	[101]
κ-TRTX-Tb1b	spider	35	3	*—*	2 [8]	12 [+2]	8.32	*—*	[101]
μ-DGTX-Dc1a	spider	56	4	++	4 [16]	19 [+1]	7.71	2	[102]
µ-TRTX-Hhn1a	spider	35	3	+	2 [9]	9 [+4]	8.65	1	[103]
µ-TRTX-Hhn2a	spider	33	3	++	3 [7]	9 [+5]	8.63	1	[104]
µ-TRTX-Hhn2b	spider	33	3	++	3 [9]	9 [+3]	8.64	1	[105]
π-TRTX-Pc1a	spider	40	3	+++	1 [8]	16 [+4]	8.66	1	[20]
τ-TRTX-Pc1c	spider	34	3	*—*	3 [10]	11 [−3]	4.48	*—*	[106]
ω-CNTX-Pn4a	spider	55	6	++	2 [12]	15 [+5]	8.3	1	[107]
APETx2	sea anemone	42	3	+	5 [11]	6 [+2]	8.32	1	[8]
OD1	scorpion	65	4	*—*	4 [21]	15 [+1]	7.67	*—*	[108]
TxIA	conesnail	17	2	+++	1 [2]	5 [−1]	4.65	1	[109]
MVIA	conesnail	32	3	*—*	3 [12]	4 [−2]	4.03	*—*	[110]
κ-SLTPX-Ssm1a	centipede	52	3	+++	2 [11]	21 [+1]	6.46	1	[111]
κ-SLTPX-Ssm2a	centipede	31	3	++	2 [8]	7 [+5]	8.33	3	[111]
µ-SLPTX-Ssm1a	centipede	32	2	+	3 [9]	12 [+2]	7.91	3	[111]

Venom peptides with molecular weight (MW) ranging from 2000–8000 Da and with 2–6 disulfide (SS) bonds have been expressed from a phylogenetically diverse range of venomous animals. ^1^Yield: +++, >5 mg/litre; ++, 1–5 mg/litre; +, 0–1 mg/litre; —, no correctly folded protein obtained; ^2^Calculated using ProtParam (http://web.expasy.org/protparam/accessed 20130220); ^3^Number of disulfide isoforms evident in final RP-HPLC purification step. N/A  =  not available.

In the sections below, each of the 9 steps in this protocol has been divided into three sections: a discussion of what options are available, an explanation of what we do, and finally, based on our experience, what we recommend is the optimal approach.

### Step 1 – What vector should I use for expressing disulfide-rich peptides?

#### What can you do?

Vector design is potentially the most important step in the successful expression of any protein/peptide of interest. There are countless choices when it comes to *E. coli* expression vectors and the selection is dependent upon numerous parameters, including the conditions under which the protein/peptide will be induced and purified. Commercially available expression plasmids are an attractive starting point as they offer pre-optimized solutions for expression in specific strains and with certain fusion tags that aid in peptide/protein purification. Vector design or modification of an existing vector can help tailor the components for individual cases. Targeting the construct to the periplasm involves the insertion of a periplasmic export sequence (or signal sequence) into the vector, such as the *MalE* signal sequence [21].

The addition of one or a combination of fusion tags can have a positive (or, if chosen poorly, negative) influence on the final yield, solubility and folding of the peptide of interest [22]. Popular tags include glutathione *S*-transferase (GST) [23], [24], maltose-binding protein (MBP) [25], [26], N-utilization substance A (NusA)[27], [28], FLAG™ [29], biotin acceptor peptide (BAP) [30], hexahistidine (His_6_) [31], streptavidin-binding peptide (STREP) [32], [33], solubility-enhancing tag (SET) [34], thioredoxin A, and calmodulin-binding peptide (CBP) [35], [36]. Among these, MBP has emerged as the preferred solubility tag for a range of diverse proteins. Although MBP is a periplasmic protein and is therefore particularly suited for such expression systems it has also found broad utility in cytoplasmic expression systems [26], [37]. An alternative (and counter-intuitive) approach is to introduce a tag that reduces the solubility of the fusion protein, such as the very insoluble ketosteroid isomerase tag that directs the expressed protein into insoluble inclusion bodies [38]. This approach is particularly useful when the protein in question is toxic to the host when soluble and properly folded. It is in these cases more common to include a cleavage site that can be used under denaturing conditions such as cyanabromide cleavage after a methionine residue [39].

In the more common case of a soluble fusion tag it is desirable to remove the tag under native conditions so it does not interfere with downstream applications. Tobacco etch virus (TEV) protease is commonly used to cleave fusion proteins because of its high selectivity [40]; its canonical seven-residue recognition site is ENLYFQ/G, with cleavage prior to the C-terminal Gly residue. Thus, a potential disadvantage of TEV protease is that it leaves a vestigial Gly residue at the N-terminus of the recombinant protein/peptide. Fortunately, detailed studies of the specificity of TEV protease [40] have revealed that it can accommodate a wide range of amino acids in the P1' site, although it prefers short-chain amino acids (Ser, Ala, Gly). Other amino acids can be accommodated but at the expense of cleavage efficiency [40]. In most cases, however, it is possible when designing a plasmid to make the last residue of the TEV protease recognition site coincide with the first residue of the native protein/peptide sequence so that TEV protease cleavage of the recombinant fusion protein yields the native sequence with no vestigial N-terminal residues. Another potential disadvantage of TEV protease is that the addition of a TEV protease cleavage site can reduce the solubility of the expressed protein [41] although we have not found this to be an issue with expression of venom peptides.

Human rhinovirus endoprotease (PreScission™) is a potential alternative to TEV protease. It is highly specific, with an 8-residue recognition site, but it leaves two vestigial N-terminal residues (Gly-Pro) that, in contrast to TEV protease, cannot be substituted with other amino acid residues [42]–[44]. Thrombin is commonly used to remove fusion protein tags, but it leaves a vestigial Gly-Ser at the N-terminus of the protein/peptide that cannot be replaced with other amino acid residues. Moreover, its six-residue recognition site provides less specificity than with TEV and PreScission™ proteases and consequently there have been reports of non-canonical cleavage by thrombin [44], [45]. Finally, Factor Xa and enterokinase can be used to generate native N-termini after digestion because their primary specificity determinants are N-terminal to the scissile bond. However, these protease have short 4/5-residue recognition sequences and thus, like thrombin, they can cleave at non-canonical sites [44], [46].

#### What do we do?

**Figure 1:**
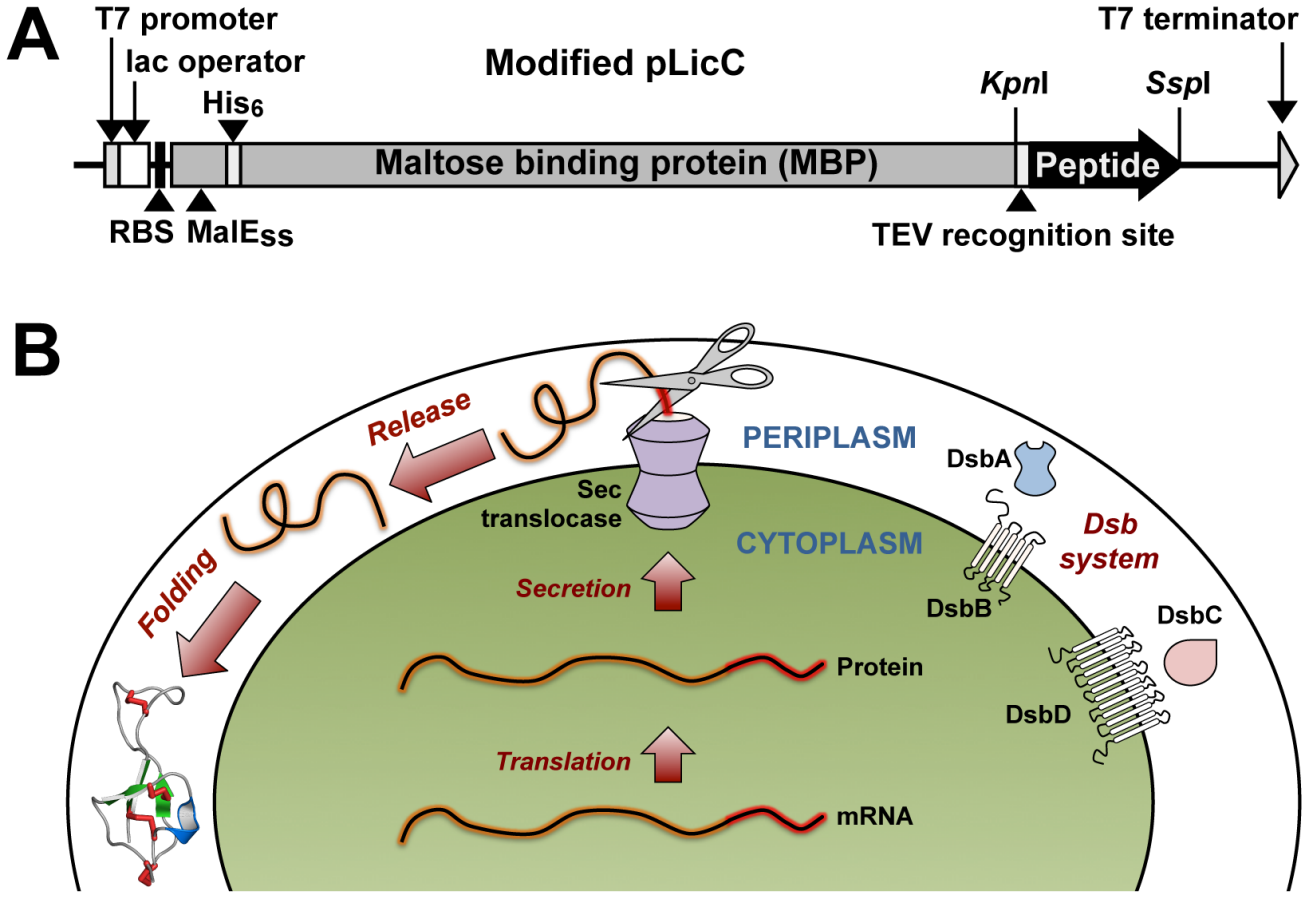
Design of expression vector. (**A**) Schematic representation of the pLic-MBP expression vector using for periplasmic expression of disulfide-rich peptides in *E. coli*. The coding region includes a MalE signal sequence (MalE_SS_) for targeting the fusion protein to the periplasm, a His_6_ tag for affinity purification, a MBP fusion tag to aid solubility, and a codon-optimised gene encoding the target peptide, with a TEV protease recognition site inserted between the MBP and target-peptide coding regions. The locations of key elements of the vector are shown, including the upstream ribosome binding site (RBS), T7 promoter, lac operator, and key restriction sites. (**B**) Schematic of the periplasmic expression system for production of disulfide-rich peptides in *E. coli*. After translation, the fusion protein is transported to the periplasm via the Sec translocase system [89]. The MalE signal sequence (red tube) is removed during this process, releasing the fusion protein (orange tube) into the periplasm where the Dsb machinery (DsbA, DsbB, DsbC, and DsbD) can assist with disulfide-bond formation.

A synthetic gene encoding the venom peptide of interest is produced by Geneart AG (Regensburg, Germany), who utilize multi-parameter algorithms to optimize codon usage to obtain high levels of protein expression [47]. The venom-peptide gene is subsequently cloned into a variant of the pLic-MBP expression vector [48]. This vector encodes a MalE signal sequence (MalE_ss_) for periplasmic export [49], a His_6_ tag for affinity purification, a maltose binding protein (MBP) fusion tag to aid solubility [26], and a TEV protease recognition site directly preceding the codon-optimised venom-peptide gene (Fig. 1A). The plasmid is transformed into the protease-deficient *E. coli* strain BL21(λDE3) and expression of the venom-peptide gene is induced with β-D-1-thiogalactopyranoside (IPTG). This leads to export of the fusion protein to the periplasm where the machinery for disulfide-bond formation is located (Fig. 1B).

#### What do we recommend?

We have had considerable success in producing a wide variety of disulfide-rich venom peptides using this variant of the pLic-MBP expression vector (see Table 1) and consequently we recommend trialling this vector system before any other. If possible, engineer the vector so that the C-terminal residue of the TEV cleavage site coincides with the first residue of your protein/peptide. If the N-terminal residue of your protein/peptide is predicted to give poor TEV protease cleavage [40], we recommend using an additional Gly as the N-terminal residue as it is the preferred P' residue for TEV protease and it is likely to have the least impact on the function of your protein/peptide.

### Step 2 – What are the optimal growth conditions for maximum yield of fusion protein?

#### What can you do?

The overexpression of fusion proteins mediated via a *lac*/T7 promoter allows for auto-induction [48]. Auto-induction is an attractive alternative to IPTG induction as less handling of the cultures is required and higher cell densities can be achieved, in addition to avoiding the cost of IPTG. A more conventional approach is to monitor the growth of the culture and add the inducer when an OD_600_ of 0.8–1.0 has been attained (i.e. during the log-phase of growth). Induction temperature is also an important consideration. A decrease in temperature prior to induction can limit aggregation of over-expressed protein in the cell [50], [51] and it also partially reduces the transcription of heat-shock proteases [52].

#### What do we do?

**Figure 2:**
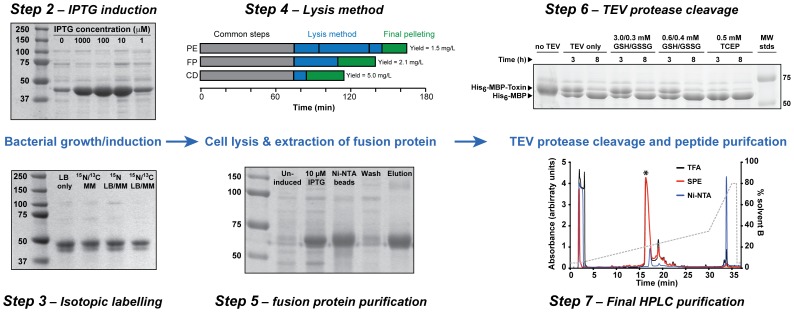
Workflow for obtaining a high yield of recombinant venom peptide. Coomassie stained SDS-PAGE gels illustrating various optimization steps. The molecular mass of the standards (in kDa) are indicated on the left or right of each gel. **Step 2**: The effect of increasing concentrations of IPTG on the level of fusion protein expression. Note that as little as 10 µM ITPG is required for induction of fusion protein expression. **Step 3**: Yields of isotopically labelled fusion protein obtaining using LB medium, minimal medium (MM), and using the dual media protocol (LB/MM). **Step 4**: Comparison of the yield of venom peptide obtained when cell pellets were obtained via periplasmic extraction (PE), French press (FP), or a constant-pressure cell disruptor (CD). **Step 5**: Ni-NTA purification of fusion protein. **Step 6**: Effect of various redox buffers on the efficiency of TEV protease cleavage of the MBP-venom peptide fusion protein. **Step 7**: RP-HPLC chromatograms comparing the efficacy of three different methods for removing His_6_-tagged fusion protein (MBP) and TEV protease prior to the final peptide purification step: precipitation with 1% TFA (black); removal with a solid phase extraction (SPE) column (red); passage of the cleavage mixture through a Ni-NTA column (blue). The asterisked peak corresponds to the peptide of interest, while the dashed line shows the gradient of solvent B (0.043% TFA in 90% acetonitrile).

We use conventional IPTG induction. The *lac* promoter is highly inducible and overexpression of the fusion protein (∼45 kDa) is evident even at IPTG concentrations as low as 1 µM (see Fig. 2, step 2). Similarly high levels of expression are obtained at final IPTG concentrations of 10, 100, and 1000 µM. Uninduced cultures typically display some evidence of background (“leaky”) expression, which is well characterized for T7 promoter-based vector systems [53]. IPTG is a costly chemical and here we show that IPTG concentrations as low as 10 µM are sufficient for high levels of fusion protein expression. We have found that venom peptides generally express better and are more soluble if the temperature is lowered to 16°C prior to induction (data not shown).

#### What do we recommend?

We recommend inducing cultures at a temperature of 16°C, starting with a final IPTG concentration of 100 µM, then titrating down to the lowest IPTG concentration consistent with maximal levels of protein expression.

### Step 3 – How do I get maximum yield of ^13^C and ^15^N labelled fusion protein?

#### What can you do?

Numerous methods have been reported for increasing the yield of isotopically labelled peptide/protein. They generally fall into two main categories: growing the entire culture in minimal medium (MM) [54] or initial growth in rich medium before transferring to MM (which we term the “*dual media approach*”) [55]. Auto-induction is also a possiblity in MM but it necessitates use of costly ^13^C-labelled glucose and glycerol [56]. MM supplements (e.g. trace metals, vitamin mixtures and commerically available enhancements) have also been shown to have a positive impact on growth and expression [57]–[59].

Not only is the dual media approach cost-effective but initial growth of the culture is faster compared to the traditional MM method. Maintaining short generation times for *E. coli* (∼30 min) by growing cells in a rich medium such as Luria-Bertani medium (LB) during the logarithmic growth phase can result in a large increase in the number of ribosomes per cell [60]. This directly correlates to higher levels of protein expression and ensures that transcription rates do not outstrip those of translation. Additionally, exchanging the medium from LB to MM prior to induction can result in an increase in expression levels by removing by-products that inhibit growth and expression [55]. Exchanging the cells to a MM volume that is one quarter less than the LB volume used to grow the culture effectively results in a four-fold increase in cell density, which can contribute to higher expression levels [55], [61]. Achieving similar cell densities using the tradition MM approach would require longer growth times.

#### What do we do?

When comparing the yield of fusion protein between cultures grown in LB, MM, or the dual-media method, we find that the final yield of fusion protein remains essentially constant regardless of the choice of labelling method (Fig. 2, step 3). The dual media approach is more labour intensive and care needs to be taken when changing media in order to avoid unintentional lysis of cells due to the mechanical forces imparted when the cell pellet is resuspended in MM. For the dual media approach, we use a D-glucose concentration of 4 g/L [55]; higher concentrations can increase the yield of recombinant protein but not in a cost-effective manner [61]. This concentration of glucose equates to only a quarter of the isotopically labelled chemicals required for the traditional MM approach. This drastically reduces the cost associated with producing sufficient labelled material for NMR-based structural analysis.

#### What do we recommend?

For producing uniformly isotopically labelled peptides we recommend the dual media protocol for both ^15^N- and ^15^N/^13^C-labelled peptides; it is as efficient as growth in LB and more cost-effective compared to the traditional MM approach. Minor variations to this approach will be required for the incorporation of more exotic isotopic labels such as ^77^Se-selenocysteine [62], which can aid in the assignment of disulfide bond connectivities [63].

### Step 4 – How do I extract my fusion protein from *E. coli* cells?

#### What can you do?

Following induction, the fusion protein can be recovered from the periplasm by selectively bursting the cell wall via osmotic shock [64] or more simply by whole-cell lysis. *E. coli* are easily lysed by several methods and for most laboratory set-ups sonication and freeze/thaw cycles are the method of choice [65]. Controlling the temperature in order to avoid overheating the sample is a problem with sonication, and this can be especially challenging when dealing with large samples. Additionally, sonication is slow and laborious. Although freeze/thawing is a gentle method of lysing *E. coli* cells, it is often incomplete, and therefore it is often used in combination with chemical agents such as lysosyme (which degrades the bacterial cell wall) and/or Bugbuster™ which introduces extra steps and expensive reagents [66], [67].

Several high-shear mechanical methods of cell lysis are also available [68]. In a French press, cells are lysed by passage through a narrow valve under high pressure (typically 150–200 MPa). This approach provides efficient lysis but it is cumbersome because of the size and weight of the apparatus, it is not well suited to processing large volumes, and the pressure cell has to be cleaned after each sample is processed. An alternative that is growing in popularity is constant-pressure cell disruption in which samples are instantly pressurized and passed at high velocity through a small orifice before being returned to atmospheric pressure.

#### What do we do?

We compared periplasmic extraction by osmotic shock with two mechanical lysis methods, namely French press and constant-pressure cell disruption. We found that the time taken to perform the lysis was inversely proportional to the final yield of protein (Fig. 2, step 4). Constant-pressure cell disruption was not only the fastest lysis method but also resulted in higher yields (5.0 mg/L culture compared to 2.1 mg/L for the French press and 1.5 mg/L for periplasmic extraction). We found that several passages through the French press were required for complete cell lysis, and it is likely that incomplete lysis is the reason for the low yield obtained here. The even lower yield obtained using periplasmic extraction may be due to premature lysis when resuspending cells in a hyperosmotic medium prior to lysis [61]. This method is also more time consuming than the mechanical methods, requiring multiple centrifugation and resuspension steps. However, this approach has been used successfully with good yields [20] and it offers the advantage of potentially easier protein purification due to the absence of cytosolic proteins. Whole-cell lysis offers the advantage of shorter processing time and higher yields. Furthermore, we have not observed shuffling of disulfide bonds due to exposure of venom peptides to the cytosolic components of the cell after lysis. This is likely due to the dilution of intracellular glutathione (GSH) and other endogenous reducing agents when cells are lysed into the buffer.

#### What do we recommend?

We recommend using a constant-pressure cell disruptor for cell lysis. In our hands, this approach achieves higher yields of fusion protein more rapidly and with less labor than other lysis methods.

### Step 5 – How do I purify my fusion protein?

#### What can you do?

Purification methods based on affinity tags offer a distinct advantage over traditional chromatographic purification methods as they enable selective purification of the protein of interest in a single step. The affinity tag chosen in Step 1 dictates which purification method will be required to “capture” the fusion protein from the cell lysate. For example, immobilized metal affinity chromatography (IMAC) is the method of choice for His_6_ affinity tags [31], while amylose resin is commonly used for affinity purification of MBP fusion proteins [21], [69]. Resins suitable for capturing specific fusion proteins are available for self-packed gravity fed columns. Alternatively, prepacked columns can be used in conjunction with a fast protein liquid chromatography (FPLC) system, which enables gradients of the mobile phase to be applied as well as real-time monitoring of the elution profile [70].

#### What do we do?

The vector we use incorporates both His_6_ and MBP fusion tags in the expressed fusion protein, and therefore IMAC and amylose affinity purification approaches could be employed individually or in tandem. We purify the His_6_-MBP fusion construct using a gravity-fed column loaded with Ni-nitrilotriacetic acid (Ni-NTA) beads (Fig. 2, Step 5); in our experience the use of amylose resin either alone or in tandem offers no significant benefit over using IMAC alone (data not shown). As evidenced in lanes 2 and 3 of Fig. 2 (Step 5), the charged Ni-NTA resin retains essentially all of the His_6_-MBP fusion protein in the cell lysate, with a negligible amount of fusion protein eluted during the column wash stage. Elution of the fusion protein with imidazole reveals the high specificity of this affinity purification approach as the eluate is devoid of other protein components that may have bound non-specifically to the resin (Fig. 2, Step 5, final lane).

#### What do we recommend?

We recommend using Ni-NTA affinity chromatography as the initial purification step because it is simple, efficient, and provides high yield and purity.

### Step 6 – How do I cleave my peptide from the fusion tag?

#### What can you do?

We focus here on TEV protease cleavage since our vector encodes a TEV protease cleavage site immediately prior to the target peptide sequence. TEV protease is a cysteine protease that utilizes a cysteine thiol as the active site nucleophile. Thus, TEV protease is only active under reducing conditions; the disulfide-linked dimer is inactive. This necessitates special caution when working with disulfide-rich peptides. The recommended reaction conditions for TEV protease cleavage include 1 mM DTT or 0.5 mM TCEP as a reducing agent, or a redox pair (3.0 mM reduced GSH/0.3 mM oxidized GSH) for proteins containing disulfide bonds [71]. However, these concentrations of GSH and oxidized GSH (GSSG) will yield a solution redox potential of –260 mV (calculated using the Nernst equation [72]). This is a very low redox potential that may result in shuffling of the disulfide bonds into non-native configurations, thus defeating the benefits of targeting the peptides to the disulfide-bond machinery in the periplasm.

The redox potential required to maintain TEV activity is unknown. However, since the disulfide-bond in the protease dimer is non-native and unlikely to be stabilising, it is likely that its redox potential is close to or higher than that of free cysteine, which is about –220 mV [73]. In contrast, the disulfide bonds of venom peptides have evolved to be major contributors to peptide stability and their redox potential is likely to be lower than that of cysteine. Thus, we use TEV cleavage buffer with 0.6 mM GSH and 0.4 mM GSSG, resulting in a redox potential of –215 mV. This milder redox buffer should maintain TEV protease in an active, reduced state while minimizing the shuffling of the pre-formed disulfide bonds in the target peptide.

#### What do we do?

To test this theory, fusion proteins were incubated with TEV protease under the recommended conditions of 0.5 mM TCEP and 3.0/0.3 mM GSH/GSSG or using the redox conditions we recommend of 0.6/0.4 mM GSH/GSSG. As can be seen from Fig. 2 (Step 6), all tested conditions had the same cleavage efficiency with the TCEP buffer yielding faster cleavage. Somewhat surprisingly, the fusion protein sample with no redox agents present was cleaved just as efficiently as when a reducing agent was added. This suggests that the dimerisation of TEV protease is slow and that sufficient amounts of monomeric TEV protease are available during the cleavage step.

#### What do we recommend?

The source of TEV protease and the buffers used during its purification will influence its activity and the redox potential required for full activation. (We produce His_6_-tagged TEV protease in-house, with final purification in a buffer containing no reducing agent). Thus, we recommend initially performing TEV cleavage in a buffer containing 0.6/0.4 mM GSH/GSSG; the redox potential of the buffer can then be increased or decreased if shuffling of disulfide bonds or inefficient cleavage is observed, respectively.

### Step 7 – How do I get rid of the affinity tags after fusion protein cleavage?

#### What can you do?

TEV protease of the fusion protein yields a mixture of the target peptide, His_6_-MBP, and in our case His_6_-TEV protease. Thus, in principle, the peptide can be purified from the His_6_-tagged MBP and TEV protease by passing the mixture through a Ni-NTA column as in the initial purification step. However, it should be noted that the backbone of a Ni-NTA bead has similar properties to a C_4_ reverse-phase (RP) HPLC column [74] and thus it will retain hydrophobic peptides. Hence, this should not be the method of choice for removing fusion tags if your peptide is hydrophobic.

Using a syringe-driven solid-phase extraction (SPE) column offers a facile alternative for separation of the large hydrophobic components (TEV protease and MBP) from the target peptide, even if the latter is somewhat hydrophobic. The disadvantage of this method is that one needs to first determine the amount and volume of organic solvent needed to elute the peptide, but not the fusion tags. Furthermore, the peptide is eluted in a solution with a high content of organic solvent that may need to be removed before the final purification step. A simple alternative approach is to precipitate out the larger proteins by addition of 1% trifluoroacetic acid (TFA). However, the disadvantage of this method is the chance of co-precipitation of the peptide with a resultant decrease in the final yield.

#### What do we do?

We compared the three methods just discussed (i.e., Ni-NTA column, SPE column, and TFA precipitation) with respect to the efficiency of MBP and TEV protease removal as well as recovery of target peptide (Fig. 2, Step 7). Although passage of the cleavage mixture over a Ni-NTA column works efficiently for some peptides [20], it can be seen from the blue trace in Fig. 2 (Step 7), that the yield of the target peptide chosen for this comparison (3.8 kDa, 3 disulfide bonds, spider peptide) is significantly lower when using the Ni-NTA column than for the two other methods tested. The SPE column and TFA extraction yielded similar amounts of the target peptide (Fig. 2, Step 7, black and red traces) and the removal of MBP and TEV protease was equally efficient.

After removal of MBP and TEV protease, a final chromatography step is typically (but not always) required to increase the purity of the target peptide to >95%, which is essential for most structural and functional analyses. We find that RP-HPLC is the most efficient method for the final purification step. The length of the alkyl chain on the RP-HPLC beads (i.e., C_18_, C_8_ or C_4_) should be chosen in inverse proportion to the hydrophobicity of the target peptide. Using a C_18_ RP-HPLC column to purify a large and highly hydrophobic peptide will result in poor yields. In our experience, RP-HPLC is almost always sufficient to produce peptides with purity >95%. Only in rare circumstances will an orthogonal chromatography step, such as ion exchange chromatography, be required to increase peptide purity to the desired level.

#### What do we recommend?

This step is highly dependent on the properties of the peptide being expressed. Unless your peptide is known to be highly hydrophobic (which would therefore exclude the Ni-NTA option), we recommend initially splitting the cleavage mixture into three batches and comparing the three methods described above for removal of the fusion tags.

### Step 8 – What is the best way to quantify the concentration of recombinant peptide?

#### What can you do?

Accurate quantitation of peptide concentration is very important for both functional and structural analyses. Quantitative amino acid analysis is the gold standard for accurately determining protein/peptide concentration as it is highly accurate and reproducible [75]. However, the procedure is complex, expensive, and generally outsourced to specialised laboratories [76]. For day-to-day experiments, simpler means of quantification are needed. Since the focus here is disulfide-rich venom peptides which typically contain multiple aromatic residues, the UV absorption at 280 nm (A_280_) provides an efficient means of concentration estimation using the molar extinction coefficient (ε) and Beer-Lambert's law (C_peptide_  =  A_280_/ε⋅l_path length_) [77]. Estimates of ε based on the amino acid sequence of the target peptide can be obtained via online predictors such as ProtParam (http://web.expasy.org/protparam accessed 20130220). A_280_ measurements are commonly being made using instruments such as the NanoDrop (ThermoScientific) due to the ease of handling and the small sample volumes required. Cuvette-based spectrophotometers employ fixed cells that require larger volumes than the NanoDrop. However, the cuvette-based approach is non-destructive and the sample can be recovered after measurement.

Dye-based methods such as the Bradford protein assay [78] and the bicinchoninic acid (BCA) protein assay [79] can also be used for protein quantification. The Bradford and BCA assays are based on the shift in absorbance when a dye (Coomassie Brilliant Blue G-250 or BCA, respectively) binds to the protein. The popular Lowry protein assay [80] is a more chemically complex assay that relies on the reaction of Cu^+^ ions, produced by oxidation of peptide bonds under alkaline conditions, with Folin-Ciocalteu reagent, leading to the oxidation of aromatic and cysteine residues; the concomitant reduction of Folin reagent can be measured at 750 nm. The major disadvantage of these methods is that the amount of dye bound, or Folin reagent reduced, depends on the sequence of the protein in an unpredictable manner. Hence, one always needs to concomitantly acquire a standard curve in order to estimate protein concentrations, and this can still lead to inaccurate concentration estimates if the property of the protein chosen as a standard differs significantly from the peptide/protein whose concentration is being measured. Moreover, in contrast with A_280_ measurement, these methods destroy the peptide/protein.

It should be noted that for most absorbance-based assays, the linear range is typically 0.1–1.0 absorbance units and hence readings outside this range will lead to inaccurate estimates of the protein concentration.

#### What do we do?

We compared peptide concentrations estimated using a BCA assay or A_280_ measurements determined using a NanoDrop or conventional spectrophotometer (Table 2) under both native and denaturing conditions (GnHCl). Overall we found that for these peptides denaturation is not generally required as the hydrophobic core of these molecules is rather small compared to large proteins, and most hydrophobic residues are solvent exposed. Traditional spectrophotometric measurement using a quartz cuvette with a path length of 1 cm produced the most accurate and reproducible measurements. The lowest protein concentration that could be reliably measured using this approach was ∼2 µM, and the standard error between readings was ∼0.5 µM. In contrast, the lowest protein concentration that could be reliably measured using the NanoDrop was 20 µM and the standard error was 5 µM.

**Table 2:** Comparison of different methods for determining protein concentration.

Instrument	Solution	Assay Method	Concentration
Plate reader	BCA reagents	Abs at 562 nm	103.9 ± 13.9
Spectrophotometer	H_2_O	Abs at 280 nm	107.0 ± 6.9
Spectrophotometer	GnHCl	Abs at 280 nm	94.2 ± 5.7
NanoDrop*	H_2_O	Abs at 280 nm	88.0 ± 8.6
NanoDrop*	GnHCl	Abs at 280 nm	98.1 ± 11.6

Concentration determination was performed using five dilutions (1∶1, 1∶2, 1∶5, 1∶10 & 1∶20) of a standard peptide solution (100 µM). The lowest dilution produced unreliable readings for all methods other than the BCA assay, and was not used for calculating the concentration or the standard deviation in those methods. Only the 1∶1 and 1∶2 dilutions produced reliable readings when using the NanoDrop; for this method, all other readings were omitted when calculating the concentration and standard deviation. * dilutions below 20 µM were unreliable and excluded.

#### What do we recommend?

If highly accurate determination of protein concentration is critical (e.g., for functional assays or circular dichroic spectropolarimetry), we recommend using a specialised laboratory for quantitative amino acid analysis. For all other purposes, we recommend determining peptide concentration via measurement of A_280_ with a conventional spectrophotometer. If the volume required is prohibitive (standard quartz cuvettes require ∼300 µL, with specialised ones requiring as little as ∼50 µL), a NanoDrop could be used, but due to the large variance between readings we recommend that the concentration estimate is based on triplicate measurements of at least at 3 dilutions of the protein.

### Step 9 – How do I acquire the best NMR data from my peptide sample?

#### What can you do?

The amount of peptide required for NMR studies will vary considerably depending on the NMR system available. Concentrations of 800–1000 µM will be suitable for most NMR instruments, with concentrations as low as 200 µM being sufficient for structural studies on a cryoprobe-equipped 900 MHz magnet [17], [19]. Standard 5-mm NMR tubes require volumes of ∼500 µL. If material is limited, one can use susceptibility-matched microtubes, which require ∼300 µL. These tubes, although more expensive, have the added benefit of minimising problems associated with convection, which can lead to poor suppression of solvent resonances.

Once a sample of sufficient concentration has been obtained, solution conditions such as pH, temperature, and ionic strength should be explored in order to maximise NMR spectral quality. The buffer should be chosen primarily to ensure that the peptide is kept at a pH where it will not aggregate during the long period of time (days to weeks) required to acquire a complete NMR dataset. In general, the sample pH should be at least one pH unit away from the isoelectric point of the peptide. High solvent conductivity can have a detrimental effect on NMR signal intensities [81] and therefore low-conductivity buffers such as MES are preferred over high-conductivity buffers such as sodium phosphate. For the same reasons, high salt concentrations should also be avoided if possible. For samples that require high salt concentrations it is worth considering alternative NMR sample tubes such as shaped tubes or 3-mm tubes to reduce sensitivity problems [82]. Finally, a screen of additives can be conducted if the sample is unstable or not monodisperse; common additives include mild detergents or salts [83]-[85]. Sample quality is most easily assessed by recording a 2D ^1^H-^15^N HSQC spectrum [17], but this requires a ^15^N-labelled sample. For more extensive screening, unlabelled samples may be used, and 1D T_2_ relaxation experiments [83] or pulsed-field gradient measurements of translational diffusion [84] can be used to estimate the aggregation state of the protein.

#### What do we do?

Fortunately, disulfide-rich peptides are commonly secreted and thus inherently very soluble. The concentration of peptides in the venom gland far exceeds the concentration used in NMR experiments and functional assays. Our standard screen therefore involves acquisition of 2D ^1^H-^15^N HSQC spectra from a ^15^N-labelled sample prepared in four different buffers: two at pH 6, one with 20 mM MES and the other with 20 mM sodium phosphate (low and high conductivity); one at pH 5 in 20 mM acetate buffer; and finally one at pH 4 in 20 mM citrate buffer. Where possible, we choose to work with one of the pH 6 buffers, as these are closest to physiological conditions.

**Figure 3:**
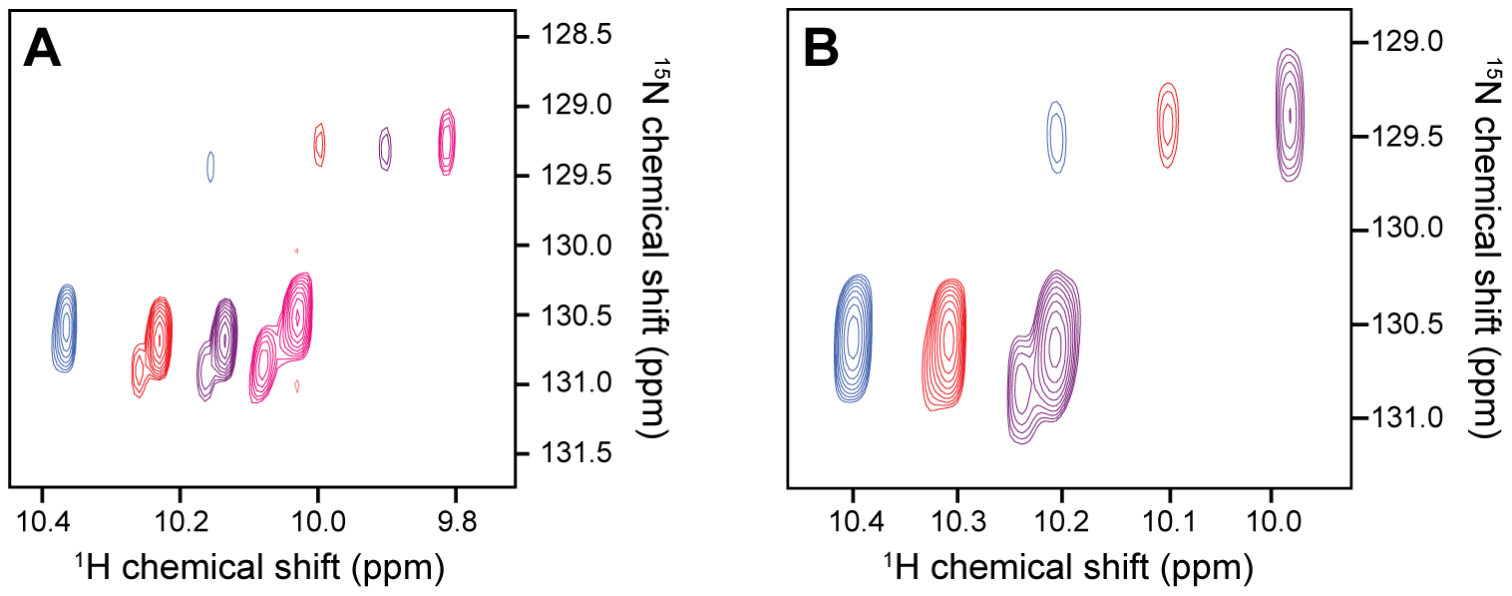
Effect of buffer, temperature, and pH on 2D ^1^H-^15^N HSQC NMR spectra of a disulfide-rich venom peptide (Step 9). (**A**) Overlays of the downfield region of 2D ^1^H-^15^N HSQC spectra of a spider-venom peptide (46 residues, 4 disulfide bonds) [90] acquired at 25°C using different buffers and pH: 20 mM MES pH 6 (pink); sodium phosphate, pH 6 (purple); 20 mM sodium acetate, pH 5 (red); 20 mM sodium citrate, pH 4 (blue). This region of the spectrum shows the sidechain ^1^H-^15^N correlation for the single Trp residue in this peptide. (**B**) Effect of temperature on the same resonance. Spectra were acquired in 20 mM citrate, pH 4 at the following temperatures: 10°C (purple); 25°C (red); 40°C (blue). At low pH and high temperature the equilibrium is shifted towards a single conformer, compared to the three conformers apparent at lower temperature.

Once the optimal buffer has been chosen, HSQC spectral quality is examined at a range of temperatures starting at 298 K and increasing up to 310 K (or higher if spectral quality is still improving with increases in temperature). In most cases, there is little difference between these temperatures, and hence the lowest temperature (298 K) is chosen for reasons of sample stability. In some instances, however, multiple peptide conformers are observed due to exchange processes such as *cis*/*trans* isomerisation of X-Pro peptide bonds [86] or conformational disulfide isomerism [87]. The presence of multiple conformers can greatly complicate NMR spectral assignment [86] and hence it is often worthwhile to try manipulating the equilibrium between conformers by subtle changes in temperature, pH, and solvent (see Fig. 3).

Finally, ^2^H_2_O (5%) and DSS [4,4-dimethyl-4-silapentane-1-sulfonic acid] (10 µM) are added for locking and referencing the NMR signals whilst additives such as EDTA, protease inhibitors, and NaN_3_ (0.02%) are often added to remove paramagnetic metal ions, prevent peptide digestion, and inhibit bacterial growth respectively.

#### What do we recommend?

We recommend preparing a ^15^N-labelled sample in order to assess peptide yields in minimal medium and to screen NMR conditions. Buffer conditions can be screened by acquiring 2D ^1^H-^15^N HSQC spectra of the sample in the four buffers described above immediately after sample preparation and after one week at room temperature. If the sample is stable after one week, prepare a ^13^C/^15^N-labelled sample in the buffer that gives the highest quality spectra; aim for a final concentration of ≥ 300 µM in 300 µL. Add 10 µM DSS and 0.02% NaN_3_, then filter the sample using a low-protein-binding Ultrafree-MC centrifugal filter (0.22 µm pore size). Details of our approach to NMR structure determination are described elsewhere [1], [17], [19].

### Conclusion – Have faith and be persistent!

We have made recommendations for *E. coli*-based production of recombinant disulfide-rich peptides based on our extensive experience with expression of disulfide-rich venom peptides. This is not intended to be a universal protocol that is guaranteed to work for all disulfide-rich peptides. The level of protein expression and the solution properties of the recombinant fusion proteins and peptides can vary greatly despite small sequence variations; small optimizations will often needed for each individual case. Nevertheless, we hope that this article serves as a useful starting point for those interested in the challenge of producing recombinant peptides or proteins with multiple disulfide bonds.

## Materials and Methods

### Steps 1 & 2: Recombinant protein production

Synthetic genes encoding the venom peptides with codons optimized for expression in *E. coli* were cloned into a variant of the pLic-MBP expression vector expression [48] by Geneart AG (Regenburg, Germany). Plasmids were transformed into *E. coli* strain BL21(λDE3) for recombinant toxin production. Cultures were grown at 37°C in LB medium supplemented with ampicillin (100 µg/mL) with shaking at 180 rpm. When the OD_600_ reached 0.8–1.0 the culture was cooled to 16°C and induced with 250 µM IPTG. Cells were harvested 12–14 h later by centrifugation for 15 min at 7741 g. Gel samples were mixed 1∶1 with 2× Coomassie blue loading dye and boiled for 5 min. 15 µL of each sample was loaded on a 12.5% SDS-PAGE gel and 7 µL of Precision Plus Protein™ standards was added to one lane to provide molecular mass markers. The gel was run for 60 min at 160 V.

### Step 3: Recombinant production of isotopically-labelled peptide

Dual media approach (LB/MM): Cultures were grown at 37°C in LB media supplemented with ampicillin (100 µg/mL) with shaking at 180 rpm. When the OD_600_ reached 0.8–1.0 the cells were harvested by centrifugation for 10 min at 3000 g. LB medium was poured off and the cell pellet resuspended carefully in M9 salts medium (22 mM KH_2_PO_4_, 90 mM Na_2_HPO_4_, 17 mM NaCl. 1.6 mM MgSO_4_. 80 nM CaCl_2_, 18 nM NH_4_Cl or ^15^NH_4_Cl, 22 mM D-glucose or ^13^C_6_-D-glucose. 2 µg/mL thiamine, 0.002% (v/v) vitamin solution, 100 µg/mL ampicillin). Cells were always resuspended in a volume of M9 medium equal to one fourth of the volume of LB used to grow the cells (e.g., 500 mL M9 medium would be used for a 2 L LB culture). The labelled culture was returned to a 37°C incubator with shaking at 180 rpm for 1 h to promote cell recovery, then cooled to 16°C and induced with 250 µM IPTG. Cells were harvested 12–14 h later by centrifugation for 15 min at 7741 g.

Minimal medium (MM) approach: Briefly, the medium composition was as follows: 16 nM FeCl_2_, 0.66 nM CaCl_2_, 0.41 nM H_3_BO_4_, 0.13 nM MnCl_2_, 0.07 nM CoCl_2_, 0.01 nM CuCl_2_, 1 mM ZnCl_2_, 1.2 mM Na_2_MoO_4_, 9.7 mM KH_2_PO_4_, 40.2 mM K_2_HPO_4_, 25 mM NaCl, 1.2 mM MgCl_2_, 275 nM K_2_SO_4_, 18 mM NH_4_Cl or ^15^NH_4_Cl, and 22 mM D-glucose or ^13^C_6_-D-glucose. Cell cultures were grown, induced, and harvested as described for the dual media approach.

### Step 4: Lysis of cells

#### Cell disruption

Whole cells were resuspended in 40 mM Tris, 400 mM NaCl, pH 8 (TN buffer) then the His_6_-MBP-toxin fusion protein was extracted from the bacterial periplasm by continuous flow cell disruption (TS Series Benchtop System, Constant Systems Ltd, Northants, UK) at a constant pressure of 30 kPa.

#### Periplasmic extraction

The His_6_-MBP-toxin fusion protein was extracted from the bacterial periplasm using osmotic shock. Briefly, the cell pellet was defrosted on ice to prevent lysis, resuspended in 30 mM Tris HCl, 2 mM EDTA, 40% sucrose, pH 7.2, then centrifuged at 17,418 for 20 min. The supernatant was discarded. The pellets were resuspended in ice-cold water and 20 mM MgCl_2_, then incubated on ice for 10 min before centrifugation as before. The supernatant was collected and enough 2 mM Tris pH 8.0, 5 M NaCl and 100% glycerol was added to give a final concentration of 20 mM Tris, 200 mM NaCl, 10% glycerol pH 8.0 (TNG buffer). The sample was then subjected to Ni-NTA affinity chromatography.

#### French Press

The His_6_-MBP-toxin fusion protein was extracted from the bacterial periplasm using a French Pressure Cell System (Biolab, Scoresby, VIC, Australia). Briefly, the cell pellet was defrosted on ice to prevent lysis, resuspended in TNG buffer, then passaged three times through a pre-cooled cell at 1,000 psi. The flow-through was collected and centrifuged at 41,399 g for 30 min. The supernatant was subjected to Ni-NTA affinity chromatography.

### Step 5 & 6: Purification and cleavage of fusion protein

The soluble lysate fraction was isolated by centrifugation at 41,399 g for 30 min and the His_6_-MBP-toxin fusion protein was captured by passing the supernatant over Ni-NTA Superflow resin (QIAGEN, Valencia, CA, USA) followed by washing with 15 mM imidazole in TN buffer to remove nonspecifically bound proteins. The fusion protein was then eluted with 500 mM imidazole in TN buffer. The imidazole was removed by centrifugal filtration (Amicon ® Ultra, Milipore), then GSH and GSSG were added to 0.6 and 0.4 mM, respectively. Approximately 40 µg of recombinant His_6_-tagged TEV protease (made in-house according to [88]) was added per mg of fusion protein. The cleavage reaction was allowed to proceed at room temperature for 12 h with shaking.

### Step 7: Purification of peptide

#### TFA precipitation

The cleaved His_6_-MBP and TEV protease were removed by precipitation with 1% v/v TFA followed by centrifugation of the precipitate for 41,399 g for 10 min. The supernatant was further purified using RP-HPLC using a Supelco apHera™ C_4_ analytical column (150×4.6 mm; particle size, 5 µm, pore size 300 Å), a flow rate of 1 ml/min and a gradient of 5 to 35% solvent B (0.043% TFA in 90% ACN) in solvent A (0.05% TFA in water) over 28 min.

#### Reapplication of cleavage mixture to Ni-NTA column

The cleaved His_6_-MBP and His_6_-TEV protease were removed from the cleavage mixture by passing it over Ni-NTA Superflow resin (Qiagen, Valencia, CA). The initial flow-through was collected, filtered and further purified using RP-HPLC as described.

#### SPE column purification

The cleavage mixture was added to solvent B to give a final concentration of 5% and filtered using a 0.22 µm syringe filter. The resulting filtrate was passed through a Maxi-Clean™ C_18_ large-pore SPE column (Grace Davison Discovery Sciences, Rowille, VIC, Australia) conditioned with methanol and equilibrated at 5% solvent B. Peptide, cleaved His_6_-MBP, and TEV protease were separated and eluted over a range of solvent B concentrations (5 mL each of 5%, 10%, 20%, 30%, 40%, 50%, 60% and 100%). Eluted samples were lyophilized and further purified using RP-HPLC as described.

### Step 8: Quantitation of peptide concentration

A ∼100 µM stock solution of a U_1_-AGTX-Ta1a was diluted 1∶1, 1∶2, 1∶5, 1∶10 and 1∶20 in water. The A_280_ absorbance was assessed in both water and guanidine hydrochloride (GnHCl). Briefly, 50 µL of the dilution stocks were made up to a final volume of 1 mL with GnHCl (6 M). A_280_ measurements were recorded using either ∼1 µL drops on a NanoDrop™ or using a 300 µL quartz cuvette in a Varian Cary 50 UV/Vis-spectrophotometer (Agilent Technologies, Mulgrave, VIC, Australia). A standard BCA assay (Thermo Scientific, Waltham, MA) was also performed on all dilutions. The absorbance at 562 nm of samples from the BCA assay were measured using a standard 96-well plate reader (PowerWave XS, Bio-Tek, VT).
